# Prior undernutrition and insulin production several years later in Tanzanian adults

**DOI:** 10.1093/ajcn/nqaa438

**Published:** 2021-03-19

**Authors:** Suzanne Filteau, George PrayGod, Andrea M Rehman, Robert Peck, Kidola Jeremiah, Rikke Krogh-Madsen, Daniel Faurholt-Jepsen

**Affiliations:** London School of Hygiene and Tropical Medicine, London, UK; National Institute for Medical Research, Mwanza, Tanzania; London School of Hygiene and Tropical Medicine, London, UK; Weill Cornell Medicine, New York, NY, USA; Weill Bugando School of Medicine, Mwanza, Tanzania; National Institute for Medical Research, Mwanza, Tanzania; Centre of Inflammation and Metabolism and Centre for Physical Activity Research, Rigshospitalet, University of Copenhagen, Copenhagen, Denmark; University of Copenhagen, Copenhagen, Denmark

**Keywords:** malnutrition-associated diabetes, HIV, Tanzania, insulin, glucose tolerance

## Abstract

**Background:**

The prevalence, pathology, and existence of malnutrition-associated diabetes remain uncertain, especially with respect to adult-acquired undernutrition.

**Objective:**

The aim was to investigate the association of prior undernutrition (low BMI, in kg/m^2^), acquired in adulthood and insulin during an oral glucose tolerance test (OGTT).

**Methods:**

We followed up 630 adults recruited 7–14 y previously for other studies. Plasma insulin was measured fasting and at 30 and 120 min during an OGTT. The main exposure was BMI measured 7–14 y prior. The main outcome of interest was plasma insulin, controlling for time during the OGTT using generalized estimating equations, and exploratory outcomes were early insulin response (relative change in insulin and glucose from 0–30 min) and relative insulin and glucose AUCs from 0 to 120 min. Current confounding factors were age, sex, BMI, HIV, socioeconomic status, and physical activity.

**Results:**

In unadjusted analyses, increasing severity of prior malnutrition was associated with lower insulin concentration. In multivariate adjusted analyses, only current BMI was a strong predictor of overall insulin concentration. Associations with prior BMI of insulin responses accounting for glucose were also seen in unadjusted but not adjusted analyses. For insulin concentration but not the outcomes accounting for glucose, there was a sex interaction with prior BMI such that only men had lower insulin if previously malnourished: insulin (pmol/L) at 120 min was 311 (95% CI: 272, 351) for prior BMI ≥18.5, 271 (95% CI: 221, 321) for prior BMI 17.0–18.5, and 237 (95% CI: 194, 297) for prior BMI <17.0; *P* = 0.03. HIV status showed limited and variable associations with insulin.

**Conclusions:**

Insulin concentration, fasting and during an OGTT, was normalized in women more than in men several years after adult malnutrition. Chronic malnutrition, as indicated by low prior and current BMI, may contribute to diabetes through low insulin secretion.

## Introduction

Most cases of diabetes now occur in low- and middle-income countries (LMICs) (1), where diabetes tends to occur at relatively low age and BMI (in kg/m^2^) (2, 3). There are heterogeneous reports of atypical diabetes in LMICs, describing an entity of malnutrition-related, insulin-deficient diabetes related to pancreatic damage (4). Although malnutrition-associated diabetes was previously included as an official category of diabetes (5), it is not a currently recognized category (4).

Despite the rising prevalence of overweight in LMICs, malnutrition as indicated by wasting remains prevalent in young children (6) and also frequently occurs in adults in association with severe infections, particularly HIV and tuberculosis. Childhood malnutrition has been identified as a risk factor for diabetes among Ethiopian adults (7). Exposure to famine during childhood was associated with increased adult diabetes in the Netherlands (8) and China (9). The Chinese study found that higher BMI or consumption in adulthood of a typically Western dietary pattern, rather than the traditional diet, increased the risk of hyperglycemia among those previously exposed to famine (9). Adult Jamaican survivors of childhood marasmus, compared with never malnourished controls, had more glucose intolerance, lower insulin secretion, and reduced insulin sensitivity (10).

Malnutrition in adulthood may also alter endocrine pancreatic function: among HIV-infected Tanzanian adults who started antiretroviral therapy (ART) when malnourished, those with the lowest fat mass either at the start of ART or 2–3 y later had the highest risk of new-onset diabetes (11). Patients with anorexia nervosa who have recovered nutritionally have been found to have impaired glucose tolerance and a delayed and lower insulin response than controls (12). The similarity between previously malnourished Tanzanians, with or without HIV or ART, and patients with anorexia nervosa who demonstrate an almost exclusively nutritional deficit lends support to the hypothesis that adult malnutrition itself has long-term effects on the pancreas with lower insulin production.

The Chronic Infections, Comorbidities, and Diabetes in Africa (CICADA) study is a cohort study investigating the burden of and risk factors for diabetes among adults in Mwanza, Tanzania. Some study participants come from our prior research on nutritional interventions for HIV or tuberculosis, and we thus know their nutritional status from several years previous when many were malnourished, as indicated by low BMI. In this secondary analysis, we used data from the cohort to investigate whether prior adult malnutrition was associated with insulin production.

## Methods

### Participants

The CICADA study is registered at clinicaltrials.gov as NCT03106480, and the cohort for this study investigating diabetes and its interactions with infections has been previously described (13). Briefly, the cohort comprised 3 groups of adults >18 y of age: *1*) participants of Nutrition, Diabetes, and Pulmonary Tuberculosis (TB-NUT; https://clinicaltrials.gov/ct2/show/NCT00311298), a nutritional supplementation trial conducted in Mwanza in 2006–2009 among tuberculosis-infected and uninfected adults (14–17); *2*) participants of Nutritional Support for African Adults Starting Antiretroviral Therapy (NUSTART; Tanzanian site), which tested the effects of adding vitamins and minerals to a lipid-based nutritional supplement on morbidity and mortality of malnourished HIV-infected participants starting ART, conducted from August 2011 to December 2013 (18), registered at the Pan African Clinical Trial registry (https://pactr.samrc.ac.za/Search.aspx) as PACTR201106000300631; and *3*) a new cohort comprising newly diagnosed HIV-infected, ART-naive participants from ART clinics in Mwanza and HIV-uninfected controls from the neighborhoods of the new HIV cohort participants. For the present analyses, we did not include the new cohort for whom we have no data on prior nutritional status. Methods and results concerning diabetes prevalence in the full CICADA cohort are published (13), and the flowchart of participants from prior studies included in this analysis is in **Supplemental Figure 1**.

### Ethics

The study was conducted in accordance with the Declaration of Helsinki of 1975, as revised in 1983. Ethical clearance was provided by the Medical Research Coordinating Committee of the National Institute for Medical Research in Tanzania and the London School of Hygiene and Tropical Medicine in United Kingdom, and consultative approval was obtained by the National Committee on Health Research Ethics in Denmark. Participants were enrolled after written informed consent, and those with diabetes and other illnesses were referred to Sekou-Toure referral hospital for care.

### HIV and tuberculosis assessment and treatment

HIV status was determined when the participants were recruited for the prior studies using 2 different rapid antibody tests: Determine HIV-1/2 (Inverness Medical Japan, Abbot) and Capillus HIV-1/2 (Trinity Biotech). Discordant samples were tested using the Uniform II Vironostika-HIV Ag/Ab Micro-ELISA system (Biomerieux BV). When recruited to CICADA, HIV-infected participants had been on ART for a median of 53 mo (IQR: 46, 102 mo). About half of the HIV-infected participants were taking azidothymidine-based regimens, and half received tenofovir-based regimens; participants taking different ART regimens had similar diabetes prevalence (13).

In TB-NUT, tuberculosis was diagnosed using a combination of clinical symptoms, sputum microscopy, and chest X-ray (15). Tuberculosis was not the focus of NUSTART, and we considered people as having tuberculosis if they were on tuberculosis treatment, given by non-study doctors, prior to starting ART (19). In both studies, patients diagnosed with tuberculosis received 6 mo of treatment according to Tanzanian and WHO guidelines. Because of this major difference in tuberculosis diagnostic methods and since all tuberculosis treatments ended many years before the current CICADA study, tuberculosis was not included in statistical analyses.

### Anthropometry

In both of the earlier studies, TB-NUT and NUSTART, and for CICADA, height and weight were measured by trained staff at enrollment using standard methods (20). Means of duplicate measures (TB-NUT) or medians of triplicate measures (NUSTART and CICADA) were used to calculate BMIs.

### Diabetes and insulin assessment

Diabetes was assessed in 2 ways: by glycated hemoglobin (HbA1c) measured on a point-of-care analyzer (HemoCue 201RT) and by a standard 2-h oral glucose tolerance test (OGTT) in which participants who had fasted overnight were given 82.5 g dextrose monohydrate (equivalent to 75 g anhydrous glucose) diluted in 250 mL of drinking water to drink within 5 min. Blood samples were collected for plasma glucose and insulin assessment at baseline and 30 and 120 min. Glucose was measured by HemoCue 501. Insulin was measured using a commercial ELISA kit (ALPCO). WHO cutoffs were used to diagnose diabetes and prediabetes (21). For HbA1c, prediabetes was defined as 5.7% to <6.5% (39 to <48 mmol/mol), and diabetes was defined as ≥6.5% (≥48 mmol/mol). For OGTT, a 120-min glucose concentration between 7.8 and 11.1 mmol/L was defined as prediabetes, and ≥11.1 mmol/L indicated diabetes.

### Statistical analyses

Analyses were conducted using Stata 16 (StataCorp). We used a significance level of 0.05 without adjustment for multiple testing as our analysis was exploratory. BMI in prior studies, the main exposure in the current analysis, was categorized as severely malnourished if BMI (in kg/m^2^) was <17.0, moderately malnourished if BMI was 17.0–18.5, and not malnourished if BMI was >18.5. At CICADA recruitment and OGTT assessment, when participants were less malnourished than in prior studies, BMI was categorized as malnourished if <18.5, normal if BMI was 18.5–25.0, and overweight if BMI was >25.

The main outcome of interest was insulin production, which we measured by plasma insulin concentrations during the OGTT conducted at CICADA recruitment. Insulin was analyzed using a generalized linear model with γ distribution and log-link function for positively skewed data and generalized estimating equations (GEEs) with an unstructured covariance matrix to account for within-person correlations of insulin concentrations at the 3 time points. Models included a time factor since fasting, early (30 min) insulin, and later (120 min) insulin indicate different phases of insulin secretion (22). In addition, to account for the fact that insulin production is related to blood glucose, we used the early 30-min insulin response (EIR), defined as (insulin_30_ – insulin_0_)/(glucose_30_ – glucose_0_), and the ratio of the total areas under the curves between 0 and 120 min of insulin/glucose (AUC_IG) as exploratory outcomes (23). EIR and AUC_IG were analyzed using general linear regression with gamma distribution and log-link function for positively skewed data.

We conducted a complete case analysis removing those missing glucose or insulin results or covariates at any of the 3 time points. We first conducted unadjusted analyses for the main exposure and key outcomes. We then investigated factors that might interact with prior BMI in associations with insulin concentrations. Because HIV can cause malnutrition and since both may be associated with increased risk of diabetes or severity of its complications (13, 24–26), we investigated whether there was an interaction between HIV status and prior BMI. Because sex is associated with many aspects of nutrition and chronic diseases, we investigated interactions of sex with prior malnutrition. We investigated the interaction between prior and current BMI based on follow-up studies of people who experienced famine in childhood, which found that risk of hyperglycemia was greater in those with subsequent high BMI or Western-style diet (9).

We then conducted adjusted analyses including current age (in 10-y bands), sex, HIV status (infected and taking ART or uninfected), current BMI (in categories listed above), current socioeconomic (SES) quintiles based on an asset index (13), and current physical activity, based on self-report using the WHO Global Physical Activity Questionnaire and its cutoff indicating adequate activity of >600 Metabolic Equivalent of Task (MET)-min per week (27). These factors were associated with either diabetes or insulin production (13) (and not shown); other lifestyle factors (smoking; consumption of fruits and vegetables, as indicated by FFQ; or alcohol) were found previously not to be associated with diabetes in the CICADA cohort (13). We assessed collinearity using scatterplots and by comparing the standard errors of adjusted models with unadjusted models; if the standard errors in the adjusted models were not larger than the unadjusted models, we assumed that no collinearity was present.

## Results

Twenty-eight of the 658 potential participants had incomplete glucose or insulin data for the 3 time points during the OGTT (Supplemental Figure 1); therefore, the analyses included 630 participants, 428 from TB-NUT and 202 from NUSTART. Compared with the full original cohorts, those recruited to CICADA differed slightly in that those still available for CICADA were older, more likely to be female, and less ill in the prior studies as indicated by CD4 count, hemoglobin, or BMI (**Supplemental Table 1**). Death during or since the previous study was a major cause of loss to follow-up, especially in NUSTART, where male sex, low CD4 count, and low BMI were risk factors for mortality (28).

The groups defined by whether or not they had been previously malnourished differed in several respects (**Table 1**). Those not previously malnourished were older, of higher SES, of higher current BMI, less likely to be HIV-infected, and less likely to have had tuberculosis in the prior study. There was no difference between groups in prevalence of diabetes and prediabetes assessed by either OGTT or HbA1c, and prevalence in all groups was quite high. A considerable number of participants had changed BMI category since their previous assessment: 20 people (7%) who were not malnourished in the prior studies had BMI <18.5 at OGTT assessment, whereas most people previously malnourished had normal BMI at this time.

**FIGURE 2:**
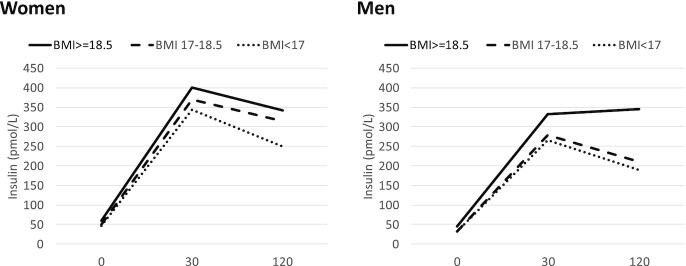
Marginal mean insulin concentrations of women and men during an oral glucose tolerance test according to prior BMI (in kg/m^2^). Values are marginal means from univariable analysis using generalized estimating equations to control for time point during the test. There were 155 female and 137 male participants with prior BMI >18.5, 86 women and 85 men with prior BMI 17.0–18.5, and 100 women and 67 men with prior BMI <17.0. Prior BMI was associated with insulin only in men at 120 min (*P* = 0.03).

**TABLE 1:** Description of CICADA population at recruitment according to prior BMI^1^

Characteristic	Prior BMI ≥18.5	Prior BMI ≥17.0 and <18.5	Prior BMI <17.0	*P* value
Number	292	171	167	
Sex, female	155 (53)	86 (50)	100 (60)	0.19
Age group, y				
18–29	20 (7)	10 (6)	14 (8)	
30–39	57 (20)	54 (32)	40 (24)	<0.001
40–49	90 (31)	69 (40)	62 (37)	
≥50	125 (43)	38 (22)	51 (31)	
HIV-infected	89 (30)	136 (80)	134 (80)	<0.001
Tuberculosis-infected in prior study^2^	118 (41)	79 (46)	92 (55)	0.02
SES quintile				
Lowest	59 (20)	52 (31)	61 (37)	
Low	48 (16)	41 (24)	37 (22)	<0.001
Middle	54 (18)	32 (19)	26 (16)	
High	57 (20)	20 (12)	22 (13)	
Highest	74 (25)	25 (15)	21 (13)	
Current BMI, kg/m^2^, mean ± SD	24.2 ± 4.9	20.2 ± 3.0	19.4 ± 2.6	<0.001
Current BMI group, kg/m^2^				
<18.5	20 (7)	54 (32)	67 (40)	<0.001
18.5–25.0	160 (55)	107 (63)	96 (57)	
>25.0	112 (38)	10 (6)	4 (2)	
Diabetes by OGTT				
Normal	141 (48)	83 (49)	92 (55)	
Prediabetes	133 (46)	84 (49)	67 (40)	0.20
Diabetes	18 (6)	4 (2)	8 (5)	
Diabetes by HbA1c				
Normal	223 (76)	129 (75)	124 (75)	
Prediabetes	43 (15)	32 (19)	28 (17)	0.65
Diabetes	26 (9)	10 (6)	14 (8)	

1 Values are presented as number (%) unless otherwise indicated. *P* values are from the χ^2^ test for categorical variables, analysis of variance for current BMI (in kg/m^2^). HbA1c, glycated hemoglobin where normal <5.7%, prediabetes 5.7–6.5%, and diabetes >6.5%; OGTT, 2-h oral glucose tolerance test where, for glucose at 120 min, normal <7.8 mmol/L, prediabetes 7.8–11.1 mmol/L, and diabetes >11.1 mmol/L; SES, socioeconomic status according to an asset index.

2 Prior tuberculosis infection data missing for 3 Nutritional Support for African Adults Starting Antiretroviral Therapy (NUSTART) participants. In Nutrition, Diabetes, and Pulmonary Tuberculosis (TB-NUT), tuberculosis was diagnosed by the study team using clinical signs and sputum tests, whereas in NUSTART, diagnoses were by nonstudy clinicians, and tuberculosis infection for study purposes was defined as taking tuberculosis medication before starting antiretroviral therapy.

**FIGURE 1:**
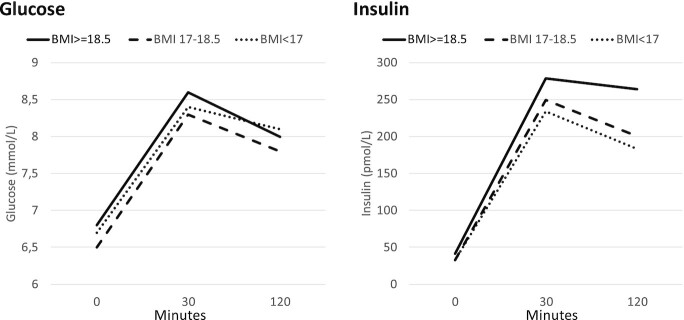
Geometric mean values of glucose and insulin during an oral glucose tolerance test according to prior BMI (in kg/m^2^). There were 292 participants with prior BMI >18.5, 171 with prior BMI 17.0–18.5, and 167 with prior BMI <17.0. Glucose differed among prior malnutrition groups only at time 0; Wald *P* = 0.005. Insulin differed among groups at all times: times 0 and 120 min, *P* < 0.001; time 30 min, *P* = 0.06.

**Figure 1** shows crude geometric mean concentrations of plasma glucose and insulin at the 3 time points in the OGTT. Glucose differed according to prior malnutrition at time 0 only (Wald *P* = 0.005), that is, glucose concentrations resolved normally during the OGTT. Insulin results are expressed as marginal means (95% CIs) from GEEs. Increasing severity of prior malnutrition, as indicated by BMI, was associated with lower plasma insulin at all time points in univariable analysis (**Table 2**). Higher current BMI was associated with higher insulin at all 3 time points and positive HIV status with lower insulin at 120 min only. Increasing age and male sex were associated with lower insulin and increasing SES with higher insulin at all time points (data not shown).

**TABLE 2:** Plasma insulin (pmol/L) during an oral glucose tolerance test according to current and prior BMI and HIV status^1^

Risk factor	*n*	Baseline	30 min	120 min
Prior BMI,^2^ kg/m^2^				
>18.5	292	53 (47, 58)	368 (336, 401)	343 (312, 375)
17.0–18.5	171	41 (37, 45)	324 (285, 364)	263 (229, 296)
<17.0	167	40 (37, 44)	312 (272, 352)	226 (205, 247)
*P* value		<0.001	0.06	<0.001
Current BMI,^3^ kg/m^2^				
>25.0	126	78 (68, 88)	520 (453, 586)	469 (412, 527)
18.5–25.0	363	42 (39, 44)	313 (290, 337)	260 (241, 279)
<18.5	141	29 (25, 32)	255 (225, 284)	207 (181, 234)
*P* value		<0.001	<0.001	<0.001
HIV status^4^				
Negative	271	47 (42, 52)	346 (314, 379)	326 (294, 358)
Positive	359	46 (42, 49)	338 (309, 366)	263 (242, 285)
*P* value		0.75	0.69	0.001

1 Values are marginal means (95% CIs) from general estimating equations using a log link and γ distribution with unstructured covariance matrix and robust standard errors adjusting for time.

2 *P* values of overall effects: time, *P* < 0.001; prior BMI, *P* < 0.001.

3 *P* values of overall effects: time, *P* < 0.001; current BMI, *P* < 0.001.

4 *P* values of overall effects: time, *P* < 0.001; HIV status, *P* = 0.098.

There was some evidence of an interaction between prior BMI and sex (Wald *P* value at time 120 min was 0.019, but joint interaction test for all times together had *P* = 0.21) in their association with plasma insulin, controlling for time using GEEs. Therefore, adjusted analyses were conducted stratified by sex (**Table 3**). Current BMI was strongly and positively associated with insulin concentration at all time points in both men and women but prior BMI only in men at 120 min. In addition to men having overall lower plasma insulin (*P* = 0.005), the reduction in insulin associated with low prior BMI appeared greater in men than in women (**Figure 2**).

**TABLE 3:** Adjusted analysis of plasma insulin (pmol/L) during an oral glucose tolerance test^1,2^

Risk factor	*n*	Baseline	30 min	120 min
Women				
Prior BMI, kg/m^2^				
>18.5	155	49 (44, 54)	356 (315, 397)	286 (250, 323)
17.0–18.5	86	51 (44, 58)	374 (310, 438)	346 (283, 409)
<17.0	100	53 (47, 60)	374 (322, 427)	293 (255, 331)
*P* value		0.57	0.87	0.20
Current BMI, kg/m^2^				
>25.0	95	77 (63, 91)	500 (405, 594)	430 (349, 511)
18.5–25.0	183	47 (43, 50)	338 (306, 369)	271 (244, 297)
<18.5	63	36 (30, 41)	310 (257, 362)	262 (209, 314)
*P* value		<0.001	0.001	<0.001
Men				
Prior BMI, kg/m^2^				
>18.5	137	41 (37, 46)	318 (280, 357)	311 (272, 351)
17.0–18.5	85	43 (37, 50)	351 (291, 411)	271 (221, 321)
<17.0	67	42 (34, 49)	325 (241, 408)	237 (194, 297)
*P* value		0.85	0.58	0.03
Current BMI, kg/m^2^				
>25.0	31	78 (63, 93)	553 (407, 700)	453 (337, 570)
18.5–25.0	180	36 (33, 39)	283 (251, 316)	244 (219, 268)
<18.5	78	24 (20, 27)	230 (193, 268)	208 (171, 244)
*P* value		<0.001	<0.001	<0.001
Combined sexes				
HIV status				
Negative	271	43 (39, 46)	323 (296, 351)	295 (269, 321)
Positive	359	49 (46, 53)	360 (330, 391)	288 (263, 312)
*P* value		0.01	0.09	0.69

1 Values are marginal means (95% CIs) from general estimating equations using a log link and γ distribution with unstructured covariance matrix and robust standard errors adjusting for time; confounders included are age, socioeconomic quintile, and whether or not physical activity was greater or less than 600 Metabolic Equivalent of Task (MET)-min per week.

2 *P* value for overall effect of sex = 0.002; *P* value for joint sex × time × prior malnutrition = 0.16; *P* value for overall effect of time = <0.001; *P* value for overall effect of prior malnutrition = 0.549; *P* value for overall effect of current BMI = <0.001; *P* value for overall effect of HIV status = 0.107.

There was no evidence of a similar interaction of prior BMI with HIV status (Wald *P* value joint test for interaction at each time was 0.92) (**Supplemental Table 2**). There was weak evidence for an interaction between prior BMI and current BMI (Wald *P* value joint test for interaction at each time was 0.07), but numbers in some subgroups divided according to prior and current BMI were too small for robust estimates (Supplemental Table 2).

For 78 participants (12%), the EIR (i.e., the ratio of the change in insulin over the change in glucose from baseline to 30 min) was negative, mainly because of a negative change in glucose (63 participants) or undefined (*n* = 8) due to zero change in glucose over 30 min. These participants were excluded from the EIR analyses. No differences were observed by age, sex, HIV status, current BMI, SES, or fasting insulin comparing those with negative or undefined EIR with those included in EIR analysis; however, those excluded from EIR analysis had a higher mean ± SD fasting glucose: 8.2 ± 2.4 mmol/L compared with 6.7 ± 1.3 mmol/L for those included, *P* < 0.001.

**Table 4** shows marginal means from unadjusted and adjusted analyses of EIR and AUC_IG from generalized linear models. For these 2 outcomes, the association with prior malnutrition was modified by current BMI (*P* for interaction = 0.03); the interaction term was included in the analysis, but separate marginal means are not presented due to very low sample sizes in some cells. There was no evidence that prior malnutrition was associated with EIR, although mean values were lower among previously malnourished people. Higher current BMI was associated with higher EIR. Being HIV-positive was associated with lower EIR in unadjusted but not adjusted analyses. For AUC_IG, prior malnutrition was associated with lower values in univariable but not adjusted analyses, current BMI was positively associated in both, and HIV status was associated in neither.

**TABLE 4:** Early insulin response and the ratio of the AUC from 0 to 120 min for insulin over the AUC for glucose^1^

Characteristic	*n*	Unadjusted	Adjusted
Early insulin response	N		
Prior BMI, kg/m^2^			
>18.5	253	317 (217, 417)	264 (181, 347)
17.0–18.5	155	217 (170, 263)	200 (153, 247)
<17.0	144	214 (144, 283)	166 (124, 208)
*P* value		0.11	0.39
Current BMI, kg/m^2^			
>25.0	111	481 (264, 698)	227 (166, 288)
18.5–25.0	318	216 (176, 255)	189 (153, 225)
<18.5	123	184 (136, 232)	205 (138, 271)
*P* value		0.001	<0.001
HIV status			
Negative	235	331 (220, 442)	219 (171, 268)
Positive	317	211 (178, 244)	194 (154, 234)
*P* value		0.02	0.33
Ratio of insulin to glucose AUC			
Prior BMI, kg/m^2^			
>18.5	292	49 (45, 53)	47 (42, 52)
17.0–18.5	171	42 (37, 47)	48 (42, 55)
<17.0	167	39 (35, 43)	46 (40, 52)
*P* value		0.004	0.82
Current BMI, kg/m^2^			
>25.0	126	68 (60, 76)	66 (56, 77)
18.5–25.0	363	40 (38, 43)	42 (38, 46)
<18.5	141	34 (30, 37)	38 (33, 42)
*P* value		<0.001	<0.001
HIV status			
Negative	271	47 (42, 51)	46 (40, 51)
Positive	359	43 (39, 46)	49 (44, 54)
*P* value		0.14	0.27

1 Early insulin response = change in insulin 0–30 min/change in glucose 0–30 min. AUC ratio = AUC insulin 0–120 min/AUC glucose 0–120 min. Original units were mmol/L for glucose and pmol/L for insulin. Values are marginal means (95% CIs). Adjusted models included all variables shown plus age, sex, socioeconomic quintile, and whether or not physical activity was greater or less than 600 Metabolic Equivalent of Task (MET)-min per week. The interaction term for EIR and current BMI, *P* = 0.03, is included. Interaction terms for AUC ratio with sex, HIV status, or current BMI and for EIR with sex or HIV status were not significant or included.

## Discussion

In univariable analyses, prior malnutrition in adults, especially for those with BMI <17.0 indicating severe malnutrition, was associated with lower plasma insulin and insulin indices during an OGTT compared with people not previously malnourished. However, in adjusted analyses, this association of prior BMI and insulin concentrations and indices was generally not significant. Men appeared at greater risk than women of low insulin concentration if they had previously been malnourished.

Other studies have shown differing risk of impaired glucose metabolism in men and women following recovery from malnutrition. Among men and women exposed to famine in Austria, men were at greater risk of developing diabetes than women after in utero or early life undernutrition (29). After exposure to famine during fetal or infant life in Nigeria, men were at higher risk of impaired glucose tolerance, as assessed by random plasma glucose, but women were at greater risk of overweight (30). This excess risk of overweight in women has also been seen after childhood malnutrition in South Africa (31). In most of Africa, as well as in the CICADA cohort (see sample sizes in Table 3), women are more likely than men to have BMI in the overweight range (32). Greater risk of overweight among women is one of multiple interacting mechanisms that contribute to sex differences in diabetes prevalence and complications in high-income situations where overweight is prevalent and prior malnutrition is rarely a concern (33). Sex differences in response to prior malnutrition associated with infections may further complicate the picture. In both Africans and Americans, HIV infection was associated with greater loss of fat-free mass in men than in women (34). Among Africans with tuberculosis-associated wasting, men gained more lean mass and women more fat mass during treatment (35, 36). Furthermore, there may be a sex differential in survivor bias after severe malnutrition: men in the NUSTART study, one component of the CICADA cohort, were more likely to die soon after recruitment to NUSTART (28), and males were less likely to survive famine in China (37). These competing sex associations of prior and current BMI and diabetes and their potential effects on both pancreas function and peripheral insulin resistance may have contributed to inconsistencies in the literature as to whether or not prior malnutrition increases the risk of later diabetes. It will be important for future studies in the field to stratify data by sex and investigate mechanisms.

HIV infection, all treated with ART in the current cohort, was generally not associated with insulin production. We previously found that any increases in diabetes risk in HIV-infected participants in the CICADA cohort were associated with inflammation-related insulin resistance in ART-naive patients, so it appears that this, rather than decreased insulin production, is key (13). Larger proportions of both the previously malnourished groups (Table 1) and the HIV-infected participants (13) had current BMI <18.5 or were in the lowest socioeconomic quintile compared with the other groups. In a previous study, HIV-infected Ethiopian adults not taking ART had lower plasma insulin both fasting and during the OGTT than in our study, but it is notable that BMI in that group was lower than in the CICADA cohort (38). Thus, it seems that associations of low prior BMI, in most cases in the CICADA population in association with HIV or tuberculosis, with low plasma insulin could be due to prolonged undernutrition or other factors associated with poverty. This is an important area for further research since both food insecurity and environmental enteropathy remain common in much of Africa (39).

We compared several different markers of insulin production—plasma insulin during fasting, both early and later in the OGTT response, the EIR, and the area under the curve in relation to glucose from baseline to 120 min—and all gave similar overall results. This strengthens confidence that the results reflect the underlying metabolism. We found differences in insulin concentrations according to several factors, but the values remained within the normal ranges (40), indicating no profound impairment in insulin production or secretion, as found in type 1 diabetes. However, a low insulin secretion capacity could increase risk of developing diabetes if challenged by insulin resistance from unhealthy diets or decreased physical activity as seen during the nutrition transition (41). We note that people with high BMI in our cohort had higher insulin production but unchanged glucose disposal during the OGTT, which could signify insulin resistance. It is likely that we see a U-shaped relation whereby both high and low BMI contribute to the increasing prevalence of diabetes in countries undergoing the nutrition transition. We are not certain why we found a higher proportion of participants with negative or undefined (due to no change in glucose) values of EIR compared with proportions found by others (23, 42). The higher baseline glucose in this group suggests some participants may not actually have been fasting.

Major strengths of the study included its large sample size and assessment of insulin responses by several methods. A major limitation was that the CICADA cohort was not randomly selected from the local population, which limits our ability to generalize. Furthermore, there were high mortality rates in the previous studies from which participants were recruited, especially NUSTART, in which both low BMI and male sex were risks for mortality (18, 28), so our data have a survivor bias; although our adjusted models did control for these and other factors, there remains the possibility of residual confounding. We did not adjust for multiple testing in our exploratory analyses, so it would be important to investigate these outcomes further in future studies. Finally, we lack data on prior malnutrition in utero or childhood, both of which may affect diabetes risk, in the participants (7, 9, 10).

In conclusion, prior malnutrition alone is not a major driver of impaired insulin production but may contribute to it, especially in men. However, chronic undernutrition is associated with low insulin production, and men who experienced severe malnutrition may develop persistent low insulin production even if their weight returns to normal. In people with HIV, chronic malnutrition may explain in part why diabetes is sometimes more prevalent among HIV-infected than uninfected people in addition to other factors such as chronic inflammation and immune activation. In Tanzania, where current and prior undernutrition is common, more attention needs to be paid to insulin production in the clinical management of diabetes. If there is a U-shaped relation between BMI and diabetes, and the pathophysiologic pathway is completely different for low and high BMI (insulin secretion compared with resistance), this may have a major impact when treating diabetes since in Africa, the usual first-line treatment, metformin (2), does not address a key problem of reduced insulin secretion.

## Supplementary Material

nqaa438_Supplemental_File

## Data Availability

Deidentified data described in the manuscript can be made available upon request to the authors and pending acceptance of a data transfer agreement, according to ethical and other approvals of the CICADA study.

## Notes

This Chronic Infections, Comorbidities, and Diabetes in Africa study was funded by the Ministry of Foreign Affairs of Denmark and administered by the Danida Fellowship Centre (grant 16-P01-TAN). SF, GP, and DF-J received funding from the UK Medical Research Council (MRC), grant MR/T006285/1. AMR is also funded by the UK MRC and the UK Department for International Development (DFID) under the MRC/DFID Concordat agreement, which is also part of the EDCTP2 program supported by the European Union, grant MR/R010161/1. RP is funded from the NIH (TW010281 and MH118107).

Supplemental Figure 1 and Supplemental Tables 1 and 2 are available from the “Supplementary data” link in the online posting of the article and from the same link in the online table of contents at https://academic.oup.com/ajcn/.

Abbreviations used: ART, antiretroviral therapy; AUC_IG, ratio of insulin to glucose AUC from 0 to 120 min; CICADA, Chronic Infections, Comorbidities, and Diabetes in Africa; EIR, early insulin response; GEE, generalized estimating equation; HbA1c, glycated hemoglobin; LMIC, low- and middle-income country; NUSTART, Nutritional Support for African Adults Starting Antiretroviral Therapy; OGTT, oral glucose tolerance test; SES, socioeconomic status; TB-NUT, Nutrition, Diabetes, and Pulmonary Tuberculosis.
