# Supplementary material for: Prior undernutrition and insulin production several years later in Tanzanian adults
Source: Am J Clin Nutr. 2021 Mar 19;113(6):1600–8. doi: 10.1093/ajcn/nqaa438 (PMC8168356; doi:10.1093/ajcn/nqaa438)
Supplement: nqaa438_Supplemental_File [file nqaa438_supplemental_file.docx]

**Prior malnutrition and insulin production several years later in Tanzanian adults; Filteau et al.**

**Online Supplementary Material**

**Supplementary Table 1. Recruitment characteristics in the original studies for participants included or not included in CICADA^1,2,3^**

|  | **TB-NUT** | | | **NUSTART** | | |
| --- | --- | --- | --- | --- | --- | --- |
|  | **Included** | **Not included** | **P** | **Included** | **Not included** | **P** |
| # (%) women | 214/428 (50%) | 712/1578 (45%) | 0.07 | 127/202 (63%) | 263/505 (52%) | 0.009 |
| Age, years (mean, SD) | 37.9 (SD 12.4) | 35.1 (SD 13.0) | <0.0001 | 39.2 (SD 9.5) | 37.6 (SD 10.0) | 0.054 |
| # (%) HIV-infected | 132/428 (31%) | 585/1575 (37%) | 0.02 | 100% | 100% | - |
| BMI (# %) >=18.5 kg/m^2^  17.0-18.5 kg/m^2^  <17.0 kg/m^2^ | 285/426 (67%)  72/426 (17%)  69/426 (16%) | 999/1572 (64%)  288/1572 (18%)  285/1572 (18%) | 0.43 | 3/202 (1.5%)  100/202 (50%)  99/202 (49%) | 0  204/505 (40%)  301/505 (60%) | 0.02 |
| # (%) tuberculosis-infected ^3^ | 180/240 (75%) | 686/1010 (68%) | 0.03 | 50/199 (25%) | 110/505 (22%) | 0.34 |
| Hemoglobin, g/L (mean, SD) | 121 (SD 29) | 118 (SD 29) | 0.08 | 95 (SD 22) | 88 (SD 24) | <0.001 |
| CD4 count, cells/μL (mean, SD) | 514 (SD 385) | 493 (SD 250) | 0.28 | 131 (SD 101) | 114 (SD 105) | 0.052 |
| Socioeconomic terciles, # (%)  Low  Middle  High | 138/303 (46%)  80/303 (26%)  85/303 (28%) | 472/974 (49%)  242/974 (25%)  260/974 (27%) | 0.67 | 68/199 (34%)  72/199 (36%)  59/199 (30%) | 167/505 (33%)  163/505 (32%)  175/505 (35%) | 0.41 |
| NUSTART treatment group,  # (%) active treatment |  |  |  | 109/199 (55%) | 246/505 (49%) | 0.15 |

^1^ In CICADA there were 3 participants who were recruited to the each of the original studies but not were included in main analyses for these so are missing some data. For NUSTART the 3 were excluded because they had BMI > 18.5 kg/m^2^ which was an inclusion criterion.

^2^ TB-NUT did not collect socioeconomic data on all participants

^3^ In TB-NUT, tuberculosis diagnosis was by sputum microscopy and X-ray among 1250 who were invited for trials; in NUSTART no formal diagnosis was done and tuberculosis positivity was taken as those on tuberculosis drugs at recruitment

**Supplementary Table 2: Insulin concentrations during an oral glucose tolerance test stratified by HIV status or current BMI^1^**

| **Prior BMI** | **Stratified by current BMI** | | |  |
| --- | --- | --- | --- | --- |
|  | **Current BMI <18.5 kg/m^2^** | **Current BMI 18.5-25.0 kg/m^2^** | **Current BMI >25.0 kg/m^2^** | **P for interaction** |
| **Baseline** |  |  |  |  |
| >=18.5 kg/m^2^ | 24 (17, 30), 20 | 38 (35, 42), 160 | 78 (67, 89), 112 | 0.10 |
| 17.0 - 18.5 kg/m^2^ | 28 (23, 32), 54 | 43 (39, 48), 107 | 84 (59, 109), 10 |  |
| <17.0 kg/m^2^ | 31 (26, 36), 67 | 46 (41, 52), 96 | 61 (45, 77), 4 |  |
| **30 Minutes** |  |  |  |  |
| >=18.5 kg/m^2^ | 263 (202, 323), 20 | 288 (261, 315), 160 | 503 (435, 570), 112 | 0.46 |
| 17.0 - 18.5 kg/m^2^ | 254 (208, 301), 54 | 322 (277, 366), 107 | 727 (419, 1036), 10 |  |
| <17.0 kg/m^2^ | 253 (207, 299), 67 | 346 (288, 405), 96 | 475 (245, 704), 4 |  |
| **120 Minutes** |  |  |  |  |
| >=18.5 kg/m^2^ | 197 (117, 278), 20 | 280 (246, 314), 160 | 460 (401, 519), 112 | 0.68 |
| 17.0 - 18.5 kg/m^2^ | 217 (171, 264), 54 | 254 (220, 288), 107 | 597 (309, 885), 10 |  |
| <17.0 kg/m^2^ | 202 (168, 237), 67 | 234 (209, 259), 96 | 414 (242, 586), 4 |  |
|  |  |  |  |  |
|  | **Stratified by HIV status** | | |  |
| **Baseline** | **HIV-uninfected** | **HIV-infected** |  |  |
| >=18.5 kg/m^2^ | 50 (44, 57), 203 | 58 (49, 66), 89 |  | 0.47 |
| 17.0 - 18.5 kg/m^2^ | 39 (31, 48), 35 | 41 (36, 46), 136 |  |  |
| <17.0 kg/m^2^ | 32 (24, 41), 33 | 42 (38, 47), 134 |  |  |
| **30 Minutes** |  |  |  |  |
| >=18.5 kg/m^2^ | 362 (324, 400), 203 | 383 (321, 446), 89 |  | 0.94 |
| 17.0 - 18.5 kg/m^2^ | 315 (231, 398), 35 | 326 (282, 371), 136 |  |  |
| <17.0 kg/m^2^ | 284 (191, 377), 33 | 319 (275, 363), 134 |  |  |
| **120 Minutes** |  |  |  |  |
| >=18.5 kg/m^2^ | 345 (306, 384), 203 | 339 (285, 393), 89 |  | 0.71 |
| 17.0 - 18.5 kg/m^2^ | 299 (223, 375), 35 | 253 (216, 290), 136 |  |  |
| <17.0 kg/m^2^ | 238 (182, 294), 33 | 223 (201, 245), 134 |  |  |

^1^ Values are marginal means (95% confidence intervals) from general estimating equations including time; sample size.

**Supplementary Figure 1. Flow chart of study participants**

Known survivors not recruited due to financial constraints, N=117

Moved from study area or not found, N=1130

Died, N=291

177 died during TB-NUT, 114 since TB-NUT)

Refused, N=18

Known survivors not recruited due to financial constraints, N=44

Moved from study area or not found, N=149

Refused, N=2

Died, N=304

(195 died during NUSTART, 109 since NUSTART)

TB-NUT participants in CICADA

N=450

NUSTART participants in CICADA

N=208

Incomplete glucose or insulin data

N=6

Incomplete glucose or insulin data

N=22

Available for analysis

N=630

NUSTART cohort

(2011-2013)

N=707

TB-NUT cohort

(2006-2009)

N=2006
